# Biodiesel Production by Single and Mixed Immobilized Lipases Using Waste Cooking Oil

**DOI:** 10.3390/molecules27248736

**Published:** 2022-12-09

**Authors:** Abir Ben Bacha, Mona Alonazi, Mona G. Alharbi, Habib Horchani, Imen Ben Abdelmalek

**Affiliations:** 1 Biochemistry Department, Science College, King Saud University, Riyadh 11495, Saudi Arabia; 2 Groupe de Recherche en Environnement et Biotechnologie, Science Department, College of Rivière-Du-Loup, Rivière-Du-Loup, Québec, QC G5R 1R1, Canada; 3 Department of Biology, College of Science, Qassim University, Buraydah 52571, Saudi Arabia

**Keywords:** biodiesel, CaCO_3_, immobilized lipase, *Bacillus stearothermophilus*, *Staphylococcus aureus*, waste cooking oil

## Abstract

Biodiesel is one of the important biofuels as an alternative to petroleum-based diesel fuels. In the current study, enzymatic transesterification reaction was carried out for the production of biodiesel from waste cooking oil (WCO) and experimental conditions were optimized, in order to reach maximum biodiesel yield. *Bacillus stearothermophilus* and *Staphylococcus aureus* lipase enzymes were individually immobilized on CaCO_3_ to be used as environmentally friendly catalysts for biodiesel production. The immobilized lipases exhibited better stability than free ones and were almost fully active after 60 days of storage at 4 °C. A significant biodiesel yield of 97.66 ± 0.57% was achieved without any pre-treatment and at 1:6 oil/methanol molar ratio, 1% of the enzyme mixture (a 1:1 ratio mixture of both lipase), 1% water content, after 24 h at 55 °C reaction temperature. The biocatalysts retained 93% of their initial activities after six cycles. The fuel and chemical properties such as the cloud point, viscosity at 40 °C and density at 15 °C of the produced biodiesel complied with international specifications (EN 14214) and, therefore, were comparable to those of other diesels/biodiesels. Interestingly, the resulting biodiesel revealed a linolenic methyl ester content of 0.55 ± 0.02% and an ester content of 97.7 ± 0.21% which is in good agreement with EN14214 requirements. Overall, using mixed CaCO_3_-immobilized lipases to obtain an environmentally friendly biodiesel from WCO is a promising and effective alternative for biodiesel production catalysis.

## 1. Introduction

One of the fundamental building blocks for a nation’s economic and social development is energy. Currently, non-renewable sources supply the majority, approximately 78%, of the world’s energy needs [1]. These resources, now limited in supply, are being consumed swiftly. Immense amounts of carbon dioxide (CO_2_) and greenhouse gas emissions (GHGs) from different anthropogenic activities continue to trigger environmental pollution, environmental deterioration and global warming [2,3]. As a result, lowering these emissions is essential, making it imperative to seek alternative non-fossil fuel-based energy systems that are environmentally safe, inexpensive, sustainable, and renewable [1]. For example, switching to biodiesel from petroleum-based fuels could be a feasible way to reduce CO_2_ and other GHG emissions. Biodiesel is a sustainable, clean-burning liquid fuel that can be produced by trans-esterifying biomass sources, including used cooking oil. Because biodiesel made from ordinary foods is more expensive than regular diesel, it is critical to concentrate on looking for cheap and easily accessible food sources in order to address this issue [4]. Waste materials such as used cooking oil are viable alternatives for the manufacturing of reasonably priced and ecologically friendly biodiesel [5]. Globally, more than 15 million tons of cooking oil are generated annually; using it to produce biodiesel would lower the environmental pollution issues associated with its disposal. The production of biodiesel fuel from waste cooling oils (WCOs) is accomplished by utilizing the lipase enzymes [6]. Lipases are the most significant class of biocatalysts, which catalyze the hydrolysis of the glycerol esters in long-chain triglycerides [4,5,6,7]. They react with the triacylglycerol’s carboxyl ester linkages, releasing fatty acids and glycerol as well as the reverse reaction, synthesis of esters from fatty acids and alcohol [7].

The rapid advancement of enzyme technology has drawn much attention to microbial lipases. These latter are more valuable than those derived from plants or animals because of the wide range of catalytic activities they can perform, the ease of genetically manipulating them, their high yield production, the lack of seasonal variations, the regular supply, and the extremely high growth rates of microorganisms in economically advantageous media. Several bacterial strains, including *Pseudomonas alcaligenes*, *P. aeruginosa*, *P. fragi*, *P. fluorescens BJ-10*, *Bacillus subtilis*, and *B. nealsonii* S2MT, as well as various fungi, including *Penicillium expansum*, *P. chrysogenum*, and *Trichoderma*, are known to produce in large quantities [7].

The main obstacle to using lipase as a biocatalyst to produce biodiesel fuel is its production cost. This issue could be solved by using immobilized enzymes. Large surface area, low cost, re-useability, good chemicals, mechanical and thermal stability and insolubility are all important qualities of solid supports utilized for immobilization [8]. Additionally, immobilization makes it simple to separate the enzyme from the final product, which reduces or completely eliminates protein contamination [9,10]. The most practical and economical methods of immobilizing enzymes continue to be adsorption onto solid supports or entrapment in bio-polymer matrixes, such as calcium alginate and carbonate. It has been proposed that immobilized lipase improves mass transfer by dispersing the enzyme over a large surface area and preventing enzyme particles from aggregating [11]. Calcium carbonate (CaCO_3_), as an immobilized phase, has the advantage of being non-toxic and unreactive to chemicals [12,13]. The benefit of using such an immobilization approach is that the enzyme does not chemically interact with the polymer, perhaps preventing denaturation. In our previous study, CaCO_3_ was successfully used to immobilize *Bacillus stearothermophilus* (*B. stearothermophilus*) and *Staphylococcus aureus* (*S. aureus*) lipases [14,15]; however, it has not been applied in biodiesel synthesis so far. Interestingly, some studies demonstrated that the use of a combination of lipases significantly increase the yield biodiesel production [16,17]. Therefore, the current study aimed to investigate the effects of single and mixed CaCO_3_-immobilized lipases on biodiesel production. After optimization of experimental conditions, the characteristics of the WCO biodiesel obtained by using CaCO_3_-immobilized lipase enzymes were evaluated and compared with the international specifications. 

## 2. Results and Discussion

WCO is a hazardous waste produced at significant levels all over the world. However, discarding WCO into various ecosystems, such as water and soil, may cause dangerous environmental consequences. In addition, given its energy content, misconduct of this hazardous waste could also be considered as a loss of resources. Therefore, looking for eco-friendly and cost-effective alternative routes to manage and valorize WCO, for instance conversion into biodiesel by enzymatic transesterification, has been extensively sought and has encouraged researchers to emphasis on emerging renewable and clean energy to overwhelmed fossil fuel supplies depletion and the global warming phenomena.

The main factors affecting the transesterification process are oil to alcohol molar ratio, type and amount of the biocatalyst, incubation temperature, reaction time, free fatty acids (FFA) content and water in substrate oil as well as the stirring speed during the chemical reaction. Table 1 summarized the main characteristics of WCO including water and free fatty acid contents, saponification, iodine and acid values. The WCO was not suitable for transesterification using an alkali catalyst because the acid content was notably high (0.85 ± 0.02%). 

Furthermore, an FFA content of more than 3% results in the formation of soap and water [18]. However, if enzymatic catalysts are used, transesterification is not affected by a high FFA in the raw materials [19]. Recorded data showed that the water content of the WCO was 0.09 ± 0.002%, and levels below 0.5% are known to be suitable for biodiesel production [20,21]. Moreover, the WCO saponification value (190 ± 3 mg KOH/g) was consistent with previously reported ranges (188–207 mg KOH/g) (Table 1) [22]. Therefore, based on its studied properties, the WCO obtained after a simulated cooking of olive oil was suitable for biodiesel production using enzymatic transesterification.

**Figure 1:**
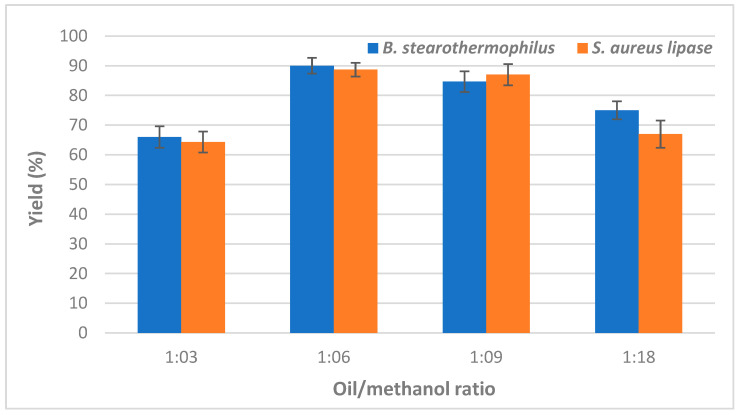
Effect of oil to methanol molar ratio on CaCO_3_-immobilized-lipase catalyzed transesterification of waste cooking oil (WCO) for biodiesel synthesis. Experimental conditions were: reaction temperature 45 °C, reaction time 24 h, water content 5%, lipase loading 1%, agitation speed 150 rpm. Experiments were performed in triplicate and error bars indicate mean ± standard deviation.

Thanks to its low-cost, methanol, the highly reactive alcohol, is commonly used for biodiesel production by transesterification reaction which requires 3 mol of alcohol per 1 mol of triacylglycerol to produce 3 mol of fatty acid methyl ester (FAME) and 1 mol of glycerol. Since it is highly recommended for base catalyst reactions to use an excess methanol: oil ratio [22], the effect of several oil/methanol molar ratios (ranging from 3:1 to 18:1) on the biodiesel yield, produced by using individual immobilized lipase, was investigated. Obtained data are illustrated in Figure 1 and clearly showed that the highest biodiesel yield values of 90 ± 2.64% and 88.66 ± 2.3% were achieved using a ratio of 6:1 by *Bacillus* and *Staphylococcus* lipases, respectively (Figure 1). 

Indeed, esterification and transesterification are reversible processes that occur simultaneously, and excess methanol is used to produce esters [23,24]. However, the lower biodiesel yields recorded for both lipases as the oil:methanol molar ratio further increases could be attributed to the denaturation and inactivation of the enzyme exposed to higher methanol concentrations [25]. Moreover, the amount of methanol has been previously reported to depend on the catalyst and substrate used [26]. For further experiments, a molar ratio of 1:6 was used.

Although most biodiesel is produced using only one lipase, some reports have demonstrated that enzyme mixtures increase biodiesel yields as the specificity of the lipase depends on the enzyme source and the substrate [27]. In the current study, *B. stearothermophilus* and *S. aureus* lipases were first individually immobilized on CaCO_3_, and the effects of immobilized single and mixed lipases were investigated at a 1% enzyme loading (Table 2). 

One can see that the lowest biodiesel yields were recorded when single immobilized *S. aureus* (88.66 ± 2.3%) or *B. stearothermophilus* (90 ± 2.6%) lipases were used. However, the highest biodiesel yield (92.66 ± 1.15%) was obtained when 1% of mixed immobilized *B. stearothermophilus* and *S. aureus* (1:1) were used. The use of mixed lipases at ratios different from 1:1 also showed lower yields (89–90%) (Table 2). This finding clearly showed that using immobilized lipase mixtures for biodiesel production was remarkably more efficient than using individual immobilized lipases suggesting that co-immobilization could be a promising alternative to optimize biodiesel production. In fact, rapid conversions could be achieved by co-immobilization of the specific enzymes within the pathway because of the localized high levels of the generated intermediates. Interestingly, the apparent lag phase is notably reduced in the presence of more enzymes in the pathway [28] which could be attributed to the synergistic activity of enzymes to break down the complex composition of WCO [29]. In the current study, the biodiesel production yield (92.66 ± 1.15%) was remarkably higher than that obtained from co-immobilization of *C. antarctica* and *R. miehei* lipases (78.4%) [30], whereas Binhayeeding et al. [16] reported a higher biodiesel production yield (96.5%) from WCO using a mixture of polyhydroxybutyrate-immobilized *C. rugosa* and *R. miehei* lipases (1:1) [16]. Similarly, the highest FAME conversion (97.2%) was obtained using a mixture of *C. antarctica* and *P. cepacia* lipases at a 25:75 ratio and microalga lipids as a substrate [27]. To our knowledge, this is the first report of biodiesel production from WCO using a combination of *B. stearothermophilus* and *S. aureus* lipases immobilized on CaCO_3_ particles at a ratio of 1:1 (50% of each enzyme) and resulting in the highest biodiesel yields. For further experiments, this ratio (1:1) was therefore selected.

**Figure 2:**
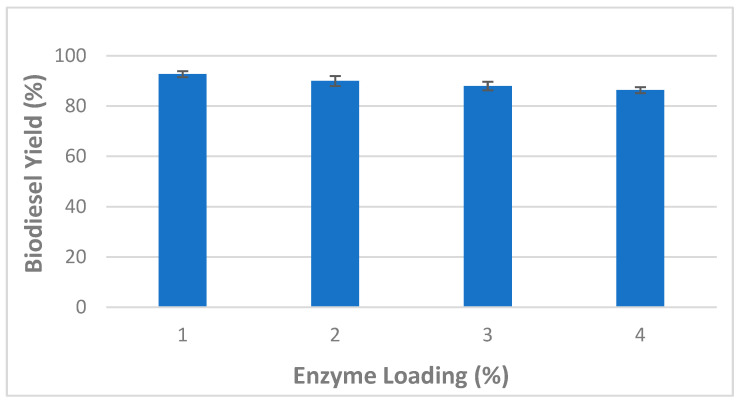
Effect of mixed CaCO_3_-immobilized lipases loading on the transesterification process of waste cooking oil (WCO) for biodiesel synthesis. Experimental conditions were: methanol/oil molar ratio: 6:1, reaction temperature: 45 °C, reaction time 24 h, water content 5%, agitation speed 150 rpm. Experiments were performed in triplicate and error bars indicate mean ± standard deviation.

The effects of enzyme loading on biodiesel production from WCOs were also investigated and obtained data are shown in Figure 2. The amount of CaCO_3_-immobilized lipase ranged from 1 to 4%. *B. stearothermophilus* and *S. aureus* lipases were mixed at a 1:1 ratio, while other factors including methanol:oil ratio (6:1), total reaction time (24 h), water content (5%), stirring speed (150 rpm) and temperature (45 °C) were kept constant. After 24 h, the highest biodiesel yield (92.66 ± 1.15%) was observed at a 1% enzyme loading (Figure 2). 

However, the gradual decrease of the biodiesel yield observed above this value (>1%) could be explained by the interference of enzymes, at higher levels, with the active sites of substrates causing their aggregation [31]. Therefore, a 1% CaCO_3_-immobilized lipase loading rate was found optimum for biodiesel production under the experimental conditions of the current study.

It is well established that the conversion of triglycerides to biodiesel is also influenced by the water level which can affect enzyme activity equilibrium value [32,33]. 

**Figure 3:**
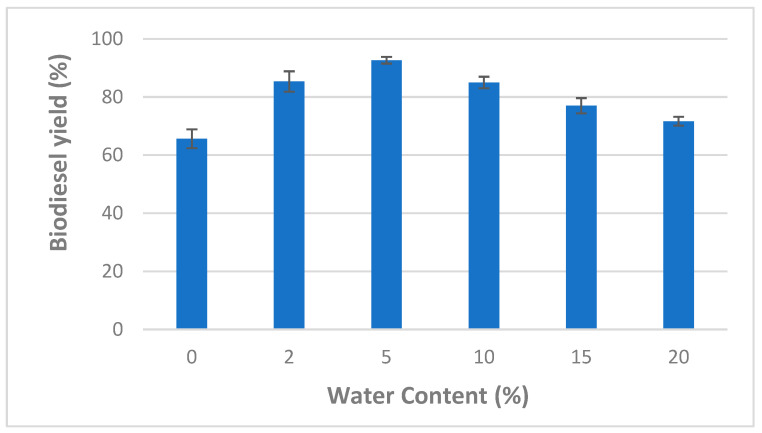
Effect of water content on mixed CaCO_3_-immobilized-lipase catalyzed transesterification process of waste cooking oil (WCO) for biodiesel synthesis. Experimental conditions were: methanol/oil molar ratio: 6:1, lipase loading 1%, reaction temperature: 45 °C, reaction time 24 h, agitation speed 150 rpm. Experiments were performed in triplicate and error bars indicate mean ± standard deviation.

Consequently, adequate water content is necessary to maintain a high enzyme activity while using organic solvents. Accordingly, the effect of different water contents (varying from 0 to 20%) on the transesterification of WCO was also investigated using CaCO_3_-immobilized lipases (at a methanol/oil molar ratio of 6:1, 1% of the enzyme mixture, after 24 h and at a temperature of 45 °C) (Figure 3).

One can see from Figure 3 that the highest biodiesel yield (92.66 ± 1.15%) was observed at a water concentration of 5%. However, at higher water content values (>5%), the biodiesel production decreased markedly. Similarly, previous studies reported that maximal lipase activity is obtained at a water content ranging from 2 to 5% [16,34,35]. In fact, lipase enzymes are able to act at the interface between the aqueous and the organic phase. Therefore, enzyme activity could be affected by increasing water contents which, in turn, could increase the interfacial area [36,37]. To optimize biodiesel production by CaCO_3_ immobilized lipases, water content of 5% was used for further experiments.

**Figure 4:**
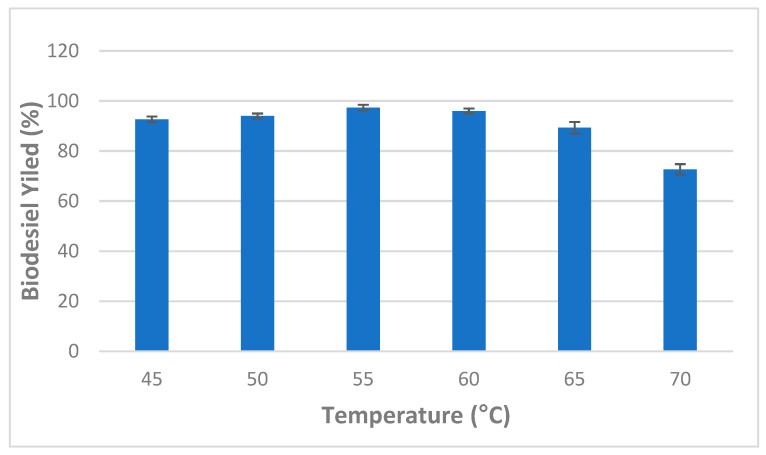
Effect of incubation temperature on mixed CaCO_3_-immobilized-lipase catalyzed transesterification process of waste cooking oil (WCO) for biodiesel synthesis. Experimental conditions were: methanol/oil molar ratio: 6:1, lipase loading 1%, water content 5%, reaction time 24 h, agitation speed 150 rpm. Experiments were performed in triplicate and error bars indicate mean ± standard deviation.

Furthermore, the effect of incubation temperature ranging from 45 to 70 °C was also investigated and obtained results are illustrated in Figure 4. It was observed that there was a significant increase in the conversion of the triglycerides to methyl esters as incubation temperature increased showing the maximum average conversion yield (97.33 + 1.15%) at an incubation temperature of 55 °C. Above 55 °C, biodiesel production yield markedly decreased due to the protein denaturation and reduced catalytic activity [38] (Figure 4). 

Binhayeeding et al. [16] reported that 45 °C was found to be the optimum temperature for the production of biodiesel using PHB-immobilized mixed *C. rugosa* and *R. miehei* lipases [16]. Similarly, Tran et al. [24] found that biodiesel production using *C. vulgaris* ESP-31 lipase decreased when temperature values increased up to 50 °C. In this study, subsequent experiments were carried out at 55 °C [24]. 

**Figure 5:**
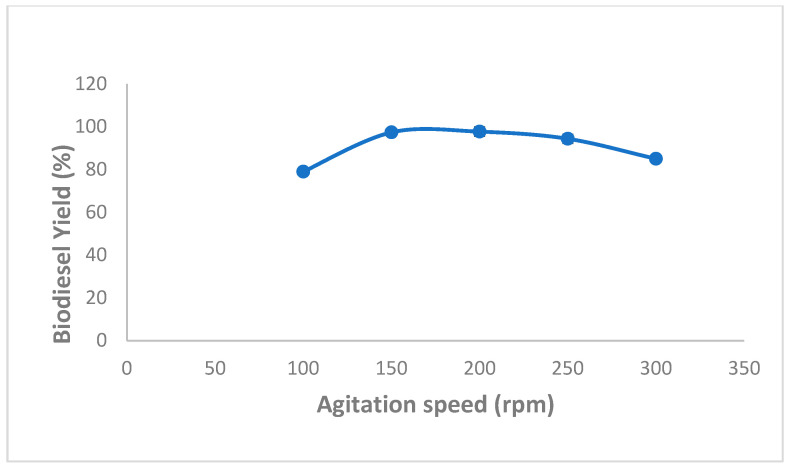
Effect of speed agitation on mixed CaCO_3_-immobilized-lipase catalyzed transesterification process of waste cooking oil (WCO) for biodiesel synthesis. Experimental conditions were: methanol/oil molar ratio: 6:1, lipase loading 1%, water content 5%, reaction temperature 55 °C, reaction time 24 h. Experiments were performed in triplicate and error bars indicate mean ± standard deviation.

Moreover, several studies demonstrated that high biodiesel production yields might be reached by immobilized enzymes using an optimized agitation speed. In this regard, the effect of various agitation speeds, ranging from 100 to 300 rpm, on biodiesel production was also evaluated and obtained data are shown in Figure 5. As we can see, the highest percentage yield of biodiesel (97.66 ± 0.57%) was attained using an agitation speed of 200 rpm (Figure 5). 

This finding could be explained by the fact that an increased agitation speed might reduce mass transfer resistance between oil and methanol and increase the reaction rate [24,39,40]. However, at an agitation speed above 200 rpm, biodiesel yields were significantly reduced, which could most likely be attributed to the agitation-associated mechanical damage to the immobilized enzymes.

**Figure 6:**
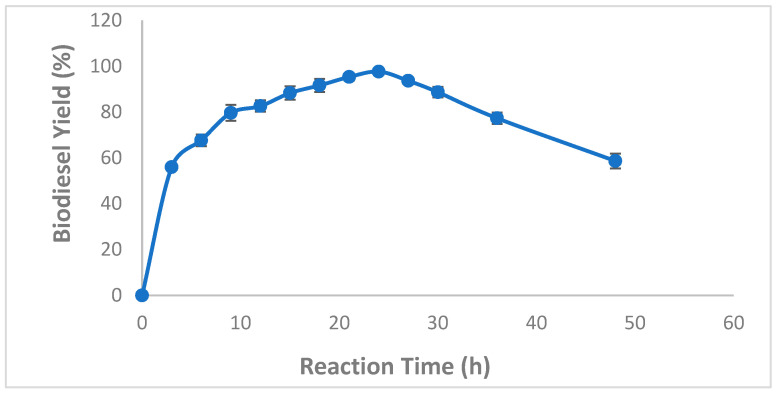
Effect of reaction time on mixed CaCO_3_-immobilized-lipase catalyzed transesterification process of waste cooking oil (WCO) for biodiesel synthesis. Experimental conditions were: methanol/oil molar ratio: 6:1, lipase loading 1%, water content 5%, reaction temperature 55 °C, agitation speed 150 rpm. Experiments were performed in triplicate and error bars indicate mean ± standard deviation.

Based on the importance of the reaction time as one of the most important factors that greatly influence the reaction equilibrium, biodiesel production from WCO using CaCO_3_-mixed immobilized *B. stearothermophilus* and *S. aureus* lipases was followed over a reaction time of 0–48 h. Results presented in Figure 6 clearly showed that as the reaction time increased, the biodiesel production yield increased to reach a maximum of 97.66 ± 0.57% after a 24 h reaction period (Figure 6). In contrast, after 24 h, The remarkable decrease in production rates was probably due to the substrate lack. Interestingly, at 1% enzyme loading, biodiesel yields reached average values up to 56 ± 1% and 67.66 ± 2.55% after 3 h and 6 h, respectively, indicating a fast kinetics of CaCO_3_-mixed immobilized *B. stearothermophilus* and *S. aureurs* lipases (Figure 6).

Our finding collaborated with previous findings demonstrating that the optimum reaction time generally ranges from 24–48 h depending on the raw material used and the enzyme source [16,18,41]. It is worthy to note that short reaction times offer several advantages such lower energy consumption, thereby facilitating large-scale production. 

**Figure 7:**
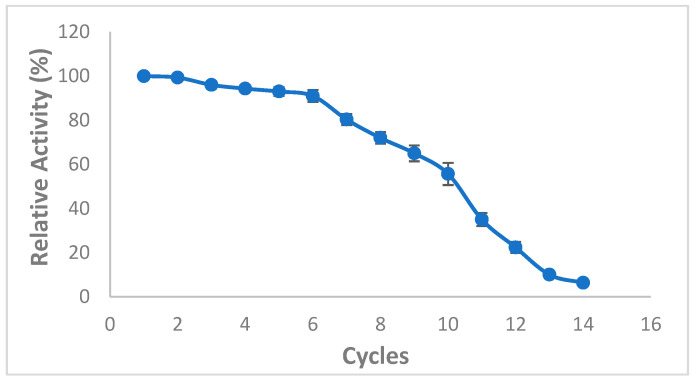
Effect of mixed CaCO_3_-immobilized-lipases reuse on the transesterification of waste cooking oil (WCO) for biodiesel synthesis. The following experimental conditions were used: methanol/oil molar ratio: 6:1, lipase loading 1%, water content 5%, reaction temperature 55 °C, reaction time 24 h, agitation speed 200 rpm. 100% is the biodiesel yield of the first reaction, while the successive reaction yields were calculated accordingly. Experiments were performed in triplicate and error bars indicate mean ± standard deviation.

The use of immobilized lipases as biocatalysts displays great potential to ameliorate the environmental sustainability and efficiency of several industrial processes, in particular biodiesel production industry. Interestingly, Zhang et al. [42] reported that CaCO_3_ adsorption technique exhibits exceptional reusable ability and mechanical rigidity for many cycles of the catalyzed reaction [42]. Thus, the reusability of immobilized lipase is of a particular importance to ascertain the feasibility of industrial-scale enzymatic biodiesel production [43]. The reusability of immobilized lipase mixture was therefore tested over 14 reaction cycles (Figure 7). First, 91 ± 2.64% of the original lipase activity was maintained even after 6 cycles, then approximately 55% of the initial lipase activity was recorded after 10 cycles indicating a promising potential reusability of the lipase mixture under the optimized experimental conditions, probably due to the enzyme interaction with the support enhancing its stability. However, after 12 cycles, the enzyme activity diminished remarkably likely due to methanol-induced conformational change and/or inactivation of lipase after numerous reaction cycles and the de-sorption, rupture of chemical bonds or erosion of the support material during the washing and recovery processes [44,45] (Figure 7).

**Figure 8:**
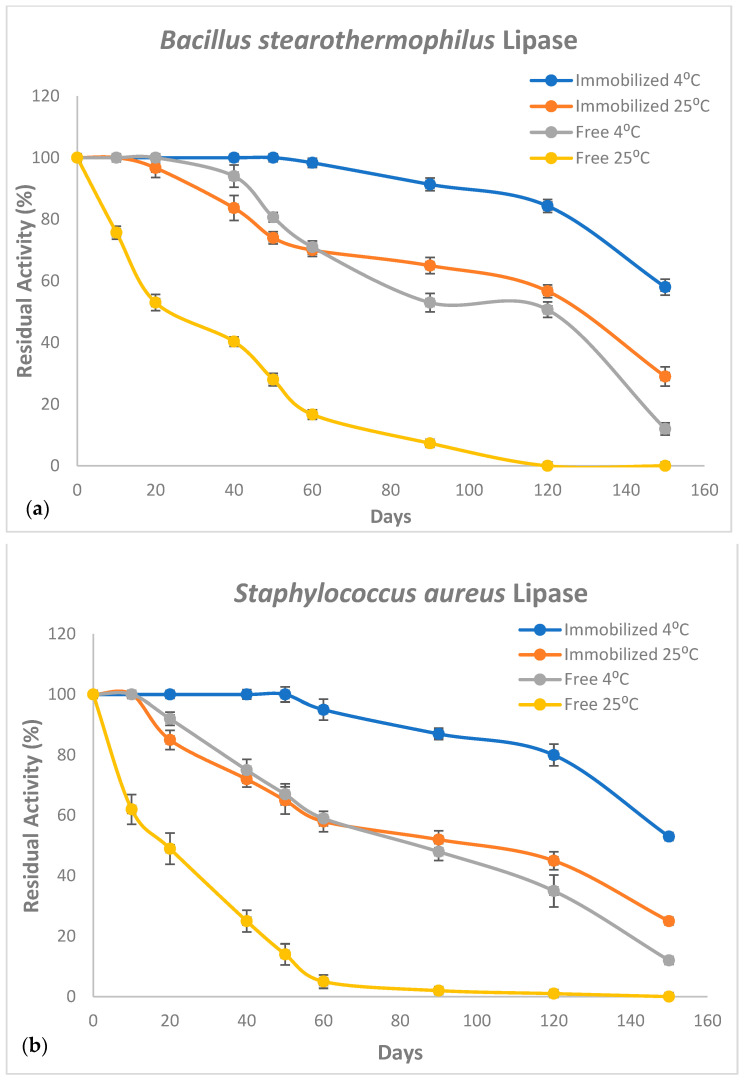
Activity retention rates (%) of CaCO_3_-immobilized and free and *B. stearothermophilus* (**a**) and *S. aureus* (**b**) lipases at 25 °C and 4 °C. Measurements were performed in triplicate and error bars indicate mean ± standard deviation.

It is well documented that the long-term storage of immobilized lipase enzymes for future use is among the most important factor for their industrial applications which is considered a significant advantage of immobilized lipases over free ones [14,16,46]. In the current report, both CaCO_3_-immobilized *B. stearothermophilus* and *S. aureus* lipases exhibited an enhanced storage capacity compared to free enzymes. Additionally, both mixed and single immobilized lipases displayed comparable storage capacities. Both immobilized lipases were almost fully active after storage for 60 days at 4 °C and then lost up to 47% activity after 150 days of storage (Figure 8). However, free enzymes retained only ~50% of their initial activities after 90 days and almost full activity was lost after 150 days of storage. Interestingly, even stored at 25 °C, both CaCO_3_-immobilized enzymes were almost fully active after 20 days compared to free lipases retaining only about 50% of their respective original activities.

GC-MS analyses of derivatized biodiesel, derivatized waste cooking oil and olive oil were carried out to check the efficacy of the biodiesel synthesis. Hence, FAMEs composition of the produced biodiesel was determined by comparing with the retention times of derivatized WCOs and of a pure FAME standard (C8–C24 standard, Sigma Aldrich, St. Louis, MO, USA). As shown in Table 3, before cooking simulation, the olive oil contained 15 fatty acids, including: myristic, pentadedecylic, palmitic, margaric, stearic, arachidic, behenic, lignoceric, palmitoleic, oleic, Cis-vaccenic, gondoic, erucic, linoleic, and linolenic acids. However, novel compounds, generated by the cooking process (caprylic acid, capric acid, lauric acid, octadecanoic acid, stearic acid allyl 9.10 epoxy, octadecanoic acid 15.16 epoxy, and 14-methylhexadecanoic acid) were observed after cooking simulation.

One can notice, in particular, the palmitoleic acid decrease against the appearance of 14-methylhexadecanoic acid together with the increase in palmitic acid (Table 3). Moreover, methylation of stearic acid likely resulted in formation of octadecanoic acid 15.16 epoxy and octadecanoic acid 9.10 epoxy probably generated from epoxidation of linolenic and linoleic acid which happens during cooking process. On the other hand, the ability of the biocatalyst to convert the oil fatty acids into methyl esters could be clearly shown from the comparison between the GC-MS analyses of derivatized biodiesel and WCOs (Table 3).

It is also worthy to note that a reduced catalytic activity was recorded as fatty acid chains length increases. The quantities of both octadecanoic acid 15.16 epoxy and octadecanoic acid 9.10 epoxy rise just seemingly, as they now weigh on an esterified smaller fraction of the initial oil. Ultimately, the difference between 100% and the calculated biodiesel yields, after 24 h, is attributed to the existence of unconverted acids, which quantity is more pronounced the longer the chains to be converted. Biodiesel produced from waste cooking oil using mixed CaCO_3_-immobilized *B. stearothermophilus* and *S. aureus*, after 24 h of synthesis, showed an amount of 0.55 ± 0.02% of linolenic methyl ester, which is in line with the EN14214. Likewise, ester content was calculated according to the modified protocol of EN14214, was found to be equal to 97.7 ± 0.21% which is in good agreement with the EN14214 as well. Furthermore, the determined iodine value (65.3 ± 1.15 g iodine/100 g) using EN 14214 was also found to be in line with the European standard. Due to the variety, complexity, and origin of the raw material, it is more essential, in this case, to validate the feasibility of WCOs oil biodiesel as fuel through comparison with the standard which is typically a key aspect. Interestingly, in the present study, the biodiesel conversions yield (97.66 ± 0.57%) is significantly high and comparable to previous reports on various oils [16,47,48,49]. However, a pre-treatment of the cooking oil may be required to improve the conversion yield and to respect the standard as well. Table 4 showing the recorded data of biodiesel characterization clearly demonstrates the feasibility of WCOs biodiesel as fuel.

## 3. Materials and Methods

### 3.1. Physicochemical Properties of WCOs

WCOs were obtained after a simulated cooking of olive oil (Sigma Aldrich-O1514, analytical grade) for 5 h at 180 °C [51]. Before the biodiesel production, WCOs physicochemical properties including: water and free fatty acid contents, acid, iodine and saponification values were determined and analyzed in accordance with EN14214. All experiments were carried out in triplicate and mean ± standard deviation (SD) values were shown in Table 1. 

### 3.2. Production and Immobilization of Bacterial Lipases

*B. stearothermophilus* and *S. aureus* lipases were produced and partially purified by two steps (precipitation with 65% ammonium sulfate followed by thermal treatment at 70 °C for 15 min) as previously described [52,53]. Then, both enzymes were immobilized on CaCO_3_ according to Rosu et al. [54] as reported in our previous works [14,15,54]. Briefly, 4500 U or 3500 U of lipase solutions from *B. stearothermophilus* and *S. aureus*, respectively, was first mixed with 1 g of the support and then kept under stirring at 4 °C for 1 h. The enzyme activity was measured in the supernatant obtained after a centrifugation (5 min at 8000 rpm). Finally, the enzyme solution filtrate was washed with double-distilled water three times and dried at room temperature for 8 h in vacuum desiccators. The resulting immobilized *B. stearothermophilus* and *S. aureus* lipases had high immobilization yields (~95%) and protein loading capacity (2.75 mg/g of immobilizing agent). Measurement of the immobilized lipase activities were carried out using a pH-stat and olive oil emulsion as a substrate under appropriate standard conditions of pH and temperature [55]. All experiments were carried out in triplicate.

### 3.3. Optimization of Biodiesel Synthesis Process from WCO 

Optimization of enzymatic biodiesel production was carried out in a 250 mL screw-capped flask. The effects of mixed and individual immobilized lipases were first examined by mixing WCO (10 g) and enzyme solution (1% wt; oil basis) while fixing the other parameters. The process was carried out for 24 h at 150 rpm, a 45 °C temperature, a methanol to oil molar ratio of 6:1 and 5% water content. After that, methanol to oil molar ratio (3:1–18:1), enzyme loading (1–4% wt), water content (0–5%), incubation temperature (45–70 °C), reaction time (1–48 h) and mixing speed (100–300 rpm) were varied. At the end of each batch, the obtained product was separated by centrifugation (20 min, 8500 rpm) and left for 1 to 2 h in a separating funnel till the resulting biodiesel was totally separated and situated in the upper layer. Next, collected biodiesel was washed twice with 20 mL hot deionized water, followed by oven-drying for 24 h at 105 °C [56]. 

The biodiesel yield (oil conversion to methyl ester) was determined as the ratio of the ester mass divided by the oil mass (%). The analysis of the fatty acids methyl esters (FAME) produced was performed by a gas chromatography–mass spectrometry (GC–MS) using a QP2010 Ultra chromatograph (Shimadzu, Japan) equipped with a model QP2010 mass spectrometer quadrupole detector. A Rxi-5Sil MS column (30 m × 0.25 mm internal diameter, 0.25 μm film thickness). GC-MS configuration: initial temperature 120 °C for 4 min, rate 1, 6.5 °C/min to 170 °C, rate 2, 2.75 °C/min to 250 °C for 9 min. Injector and detector temperatures were set 250 °C and 230 °C, respectively. Helium was used as the carrier gas. Composition of FAMEs obtained from WCOs derivatization using Methanol:BF3 method [57] was compared with the biodiesel produced and a known concentration FAME mixture. EN14214 was used to evaluate methyl ester content in the produced biodiesel [58]. Measurements were performed in triplicate.

### 3.4. Reuse of Immobilized Lipases

Immobilized enzymes’ reusability was assessed by recovering them after each transesterification reaction and utilizing them for subsequent cycles under the same conditions. The immobilized lipase was filtered at the end of each cycle, using a paper filter, then washed twice with ultrapure water and hexane and finally dried for 24 h in a desiccator prior to use. After that, both enzyme and the substrate were used again for 14 cycles under optimal experimental conditions. A total of 100% is the biodiesel yield of the first reaction, while the successive reaction yields were calculated accordingly.

### 3.5. Storage Stability

The stabilities of the immobilized mixed and individual lipases were determined by calculating the residual activity over a period of 150 days. Both free and immobilized (in wet form) enzymes were stored at 4 °C or 25 °C in a 0.2 M Tris-HCL buffer (pH 8).

## 4. Conclusions

Enzymatic catalysts have recently received increased attention in biodiesel production industry by employing immobilized lipases as biocatalysts. Herein, biodiesel production was effectively improved using a mixture of CaCO_3_-immobilized *B. stearothermophilus* and *S. aureus* lipases through the enzymatic transesterification of WCO. The best biodiesel production yield (97.66 ± 0.57%) was attained under the optimal experimental conditions: a methanol: oil ratio of 6:1, enzyme loading 1% (50% of each lipase), 1% water, reaction temperature of 55 °C for 24 h under continuous stirring speed of 200 rpm. Similarly, to previous studies, higher biodiesel yields were achieved by using mixture of immobilized *stearothermophilus* and *S. aureus* lipases than either of the lipases alone. Moreover, the characteristics of the biodiesel described in the current study were comparable to those of commercial biodiesel and in agreement with the international requirements (EN 14214). Collectively, our data demonstrated that CaCO_3_-immobilized lipases mixtures are extremely versatile, require slight amounts of biocatalyst, process a rapid production time, accelerate substrates’ conversion into products, and could be easily recycled. The interesting characteristics of enzymes described is this study as well as their biodiesel production yields open the door to using them as biocatalysts to synthesize other biodiesel such as ethyl esters or a mixture of methyl and ethyl esters as described by Yussof et al. [59] and other high value-added products using other residual materials in a context of circular bioeconomy. 

## Figures and Tables

**Table 1:** Physicochemical characterization of waste cooking oil (WCO). Values are the averages of triplicate determinations ± standard deviation (SD).

Physicochemical Properties	
Property	Average ± SD
Moisture (%)	0.09 ± 0.002
Saponification Index (mgKOH/g)	190 ± 3
Acid value (%)	0.85 ± 0.02
Free Acid Content (%)	1.35 ± 0.13
Iodine value (g I_2_/100 g oil)	68.7 ± 2.51

**Table 2:** Effect of CaCO_3_-immobilized individual and mixed lipases on the transesterification of waste cooking oil (WCO) under the following experimental conditions: enzyme loading 1% wt, 1:6 oil to methanol molar ratio, 5% water content, a 45 °C temperature reaction, a 24 h reaction time and 150 rpm agitation. Values are the mean of the three replicates ± SD.

Percentage of Immobilized Lipase Mixture		Biodiesel Yield (%)
*B. stearothermophilus*	*S. aureus*	
100	0	90 ± 2.64
75	25	89.33 ± 1.15
50	50	92.66 ± 1.15
25	75	90.33 ± 2.51
0	0	88.66 ± 2.31

**Table 3:** Fatty acid compositions (%) of olive oil from Sigma Aldrich, waste cooking oil (WCO), and produced biodiesel with of 1% of immobilized lipase mixture concentration.

Fatty Acid	Common Name	Olive Oil	WCO	Biodiesel
C8	Caprylic	-	0.27 ± 0.02	0.38 ± 0.01
C10	Capric	-	0.05 ± 0.01	0.06 ± 0.01
C12	Lauric	-	0.03 ± 0.01	0.02 ± 0.01
C14	Myristic	0.01 ± 0.02	0.01 ± 0.01	0.01 ± 0.02
C15	Pentadedecylic	0.01 ± 0.01	0.02 ± 0.01	0.02 ± 0.01
C16	Palmitic	17.00 ± 0.41	20.30 ± 0.56	25.6 ± 0.23
C17	Margaric	0.15 ± 0.01	0.08 ± 0.01	0.11 ± 0.02
C18	Stearic	2.34 ± 0.11	2.04 ± 0.20	1.48 ± 0.21
C20	Arachidic	0.38 ± 0.05	0.26 ± 0.04	0.08 ± 0.04
C22	Behenic	0.12 ± 0.04	0.25 ± 0.07	-
C24	Lignoceric	0.05 ± 0.02	0.02 ± 0.01	-
C16:1ω7	Palmitoleic	2.15 ± 0.11	1.32 ± 0.02	1.77 ± 0.03
C17:1 ω7	-	0.08 ± 0.02	0.03 ± 0.02	0.6 ± 0.02
C18:1 ω9	Oleic	56.66 ± 1.36	55.12 ± 0.25	57.2 ± 0.25
C18:1 ω7	Cis-vaccenic	2.95 ± 0.08	2.77 ± 0.05	2.90 ± 0.10
C20:1ω9	Gondoic	0.21 ± 0.06	0. 3 ± 0.07	-
C22:1 ω9	Erucic	-	-	-
C18:2 ω6	Linoleic	17.17 ± 0.47	14.06 ± 0.88	6.5 ± 0.86
C18:3 ω3	Linolenic	0.69 ± 0.05	0.39 ± 0.04	0.19 ± 0.05
	Octadenoic Acid 9.10 Epoxy	-	0.47 ± 0.01	0.75 ± 0.04
	Stearic Acid Allyl	-	0.52 ± 0.03	0.72 ± 0.03
	14-Methylhexadecanoic	-	0.55 ± 0.02	0.71 ± 0.01
	Octadecanoic Acid 15.16 Epoxy	-	0.68 ± 0.01	0.81 ± 0.02

**Table 4:** Characterization of the biodiesel produced from waste cooking oil (WCO).

Fuel Properties	Biodiesel Current Study	Biodiesel Standard [50]
Density at 15 °C	805	878
Viscosity at 40 °C	2.5	1.9–6
Flash point °C	>120	100–170
Cetane number	-	48–65
Moisture content (ppm)	Trace	0.05% max
Acid Value (mgKOH/g)	0.4	0.5
Polyunsaturated methyl esters (% m/m)	0	0
Methanol content (% m/m)	0.11	0.2

## Data Availability

All data are available in the main text.
